# Acral peeling skin syndrome in two East-African siblings: case report

**DOI:** 10.1186/1471-5945-12-2

**Published:** 2012-03-19

**Authors:** Samson K Kiprono, Baraka M Chaula, Bernard Naafs, John E Masenga

**Affiliations:** 1 Department of Dermatology, Regional Dermatology Training Center at KCMC, Box 8332, Moshi, Tanzania; 2 Stichting Tropendermatologie, Munnekeburen, The Netherlands

**Keywords:** Acral peeling skin syndrome, African, Siblings

## Abstract

**Background:**

Acral peeling skin syndrome is a rare autosomal recessive genodermatosis due to a missense mutation in transglutaminase 5. The skin peeling occurs at the separation of the stratum corneum from the stratum granulosum.

**Case presentation:**

We present a case of two siblings who developed continuous peeling of the palms and soles from the first year of life. This peeling was more severe on the soles than palms and on younger sibling than elder sibling. Peeling is worsened by occlusion and sweating.

**Conclusions:**

Sporadic cases of Acral Peeling Skin Syndrome occur in African population. There is variability in time of presentation and clinical severity even within families.

## Background

Peeling Skin Syndrome is a rare autosomal recessive skin disorder characterized by an asymptomatic superficial exfoliation due to separation of the stratum corneum [1]. It was first described in the early twentieth century [2] and broadly classified into localized and generalized forms. The generalized form is further subdivided into inflammatory (type A) and non-inflammatory (type B) [3]. Acral Peeling Skin Syndrome (APSS) is considered to be a localized variant [4]. We present a case report of two siblings with APSS.

## Case presentation

**Figure 1:**
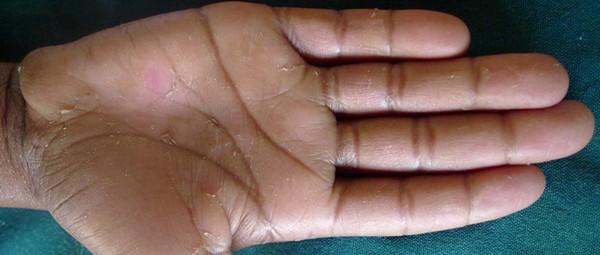
**Left hand of 13 years old boy showing peeling of the palms**.

**Figure 2:**
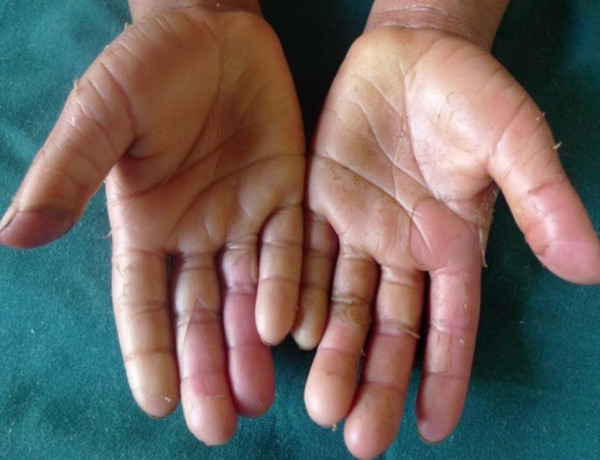
**Hands of the 6 years old girl showing whitish thickening of the palms which peel leaving behind a soft erythematous skin**.

Two siblings presented with asymptomatic peeling of the hands and the feet. The elder sibling was a 13 year old boy who developed peeling of the skin in the first year after birth. It was noticed that the peeling of the skin left a soft red skin, which progressively thickened over a period of one to two months and turning pale before peeling again in sheets (Figure 1). The younger sibling was a 6 year old girl who had peeling of the hands and feet from birth. The problem was more severe in the younger sibling than the older sibling (Figure 2). It was associated with hyperhidrosis and became foul smelling when occluded. The peeling of the skin was aggravated by sweating, prolonged exposure to water and occlusion. There was neither associated pain nor pruritus.

They came from a family of five siblings. Their parents and the other three siblings did not have a similar problem. It was a consanguineous marriage of first cousins, although no similar problem among their family members.

**Figure 3:**
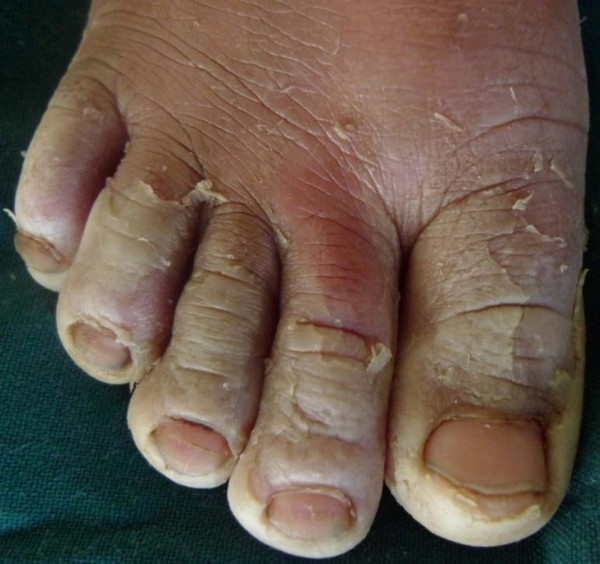
**Right foot of 6 years old girl with peeling of the feet extending to the dorsum**. It is more prominent on the toes. The toes are also macerated after wearing closed shoes.

On examination, the siblings were generally in good health with normal development. A bilateral and symmetrical peeling of the hands and the feet, worse on the palmer and plantar areas was noted. The peeling extended to the dorsal aspects of both the hands and the feet. The pale areas of the palms could be peeled with minimum pain leaving behind a soft, red skin. The peeling was thicker with features of maceration in the younger sibling (Figure 3).

**Figure 4:**
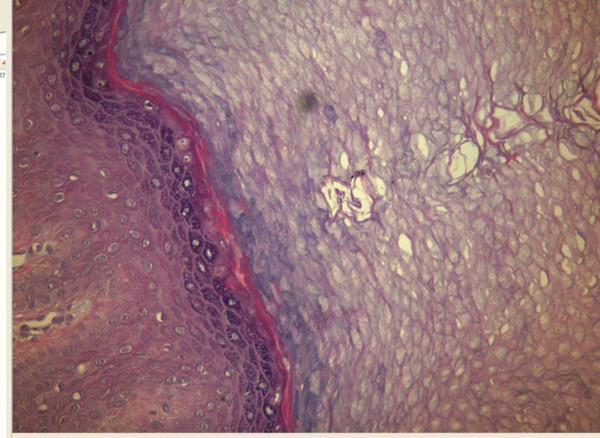
**Hematoxylin and Eosin staining of the palmer: Hyperkeratosis, acanthosis and separation of the stratum corneum from underlying stratum granulosum**.

The teeth, the hair and the nails were normal. Skin biopsy showed separation of stratum corneum from stratum ganulosum (Figure 4). Full blood picture, liver function tests and renal function tests were within normal range. A diagnosis of APSS was made and both siblings treated with topical tretinoin 0.05% cream for four months without any improvement.

## Discussion

Patients with continuous peeling of the skin were first reported as Keratolysis Exfoliativa Congenita [2]. This changed when in 1982 Levy and Goldsmith [5] described it as a peeling skin syndrome. Peeling skin syndrome is an extremely rare inherited skin disorder characterized by continual, spontaneous skin peeling (exfoliation). APSS is considered a subtype of peeling skin syndrome in which the peeling of the skin is limited to the dorsa of the hands and the feet [6,7]. The skin peeling is due to separation of the stratum corneum from the stratum granulosum [1]. Molecular studies among family members with APSS showed homozygous missense mutation in transglutaminase 5 (TGM5) [7,8]. Cassidy et al. [7] conducted a genome-wide linkage analysis and localized the genetic defect on chromosome 15 (at 15q15.2). This region contains nine genes with a small transglutaminase (TGM) gene cluster at its center. However, only TGM5 has a strong expression in the skin. The TGM5 enzyme is responsible for introducing γ-glutamyl- ε-lysine isopeptide bonds into the structural proteins [9]. The weakness of these bonds leads to a split in a region between the granular layer and the stratum corneum, which is similar to a split in APSS.

Laboratory studies in some patients with peeling skin syndrome showed abnormalities in the levels of amino acids, plasma tryptophan, serum copper, ceruplasmin and iron binding capacity [10]. Clinically, APSS is asymptomatic peeling with residual erythema for a few days that later heal spontaneously without scaring [7,8]. The onset of APSS is variable, but in the majority of the cases reported, the disease developed shortly after birth. One of our patients presented with peeling from birth and the elder sibling at one year. These patients had an earlier age of onset than the Tunisian patients who almost had a similar clinical presentation. APSS has been described to affect predominantly the dorsal aspects of the hands and the feet [7,8]. However, Hashimoto et al. [6] described a 34 year old patient with peeling of the plantar and the dorsal of the feet. Our patients had involvement of both involvement of the hands and the feet, but it was more severe on the palm and the plantar area than it was on the dorsa. The variability in the phenotypic expression of the two siblings indicates that the heterogeneity of clinical findings is not only between families, but also within family members.

The symptoms of APSS are aggravated by hot temperatures, high humidity and friction [8].

Hashimoto et al. [6] and Wakade et al. [3] demonstrated that skin hydration by soaking in water for 5-10 minutes induced blistering in their patients The slightly more severe disease in our patients may be due to excessive sweating, the use of occlusive shoes and the hot humid tropical climate. Hyperhidrosis produces a similar effect as soaking the limbs in water.

There is no effective treatment for APSS reported. Management of this condition is largely symptomatic and preventative. Emollients are useful to some patients while the use of keratolytics may enhance the shedding. Topical tretinoin was found to be ineffective in this patient. Similarly, Phototherapy, oral corticosteroids and methotrexate were found to be in effective [5]. Calcipotriol was reported to be effective in one case report [11].

## Conclusions

The two siblings from a consanguineous marriage had an earlier age of onset of asymptomatic superficial exfoliation that was worse on the palm and the plantar area. This was exacerbated by sweating and wearing closed shoes. Treatment with topical tretinoin 0.05% yielded no improvement. Currently, there is no satisfactory treatment for APSS.

## Consent

Written informed consent was obtained from the mother of the two siblings for publication of this report and any accompanying images.

## Competing interests

The authors declare that they have no competing interests.

## Authors' contributions

SK, BM, BN, JM participated investigation and diagnosis, SK and BM drafted the manuscript while BN and JM reviewed the manuscript. All authors read and approved the final manuscript.

## Pre-publication history

The pre-publication history for this paper can be accessed here:

http://www.biomedcentral.com/1471-5945/12/2/prepub
